# Discovering deaminases using AlphaFold2: a strategy to search for tool proteins for gene editing

**DOI:** 10.1038/s41392-024-01737-z

**Published:** 2024-02-07

**Authors:** Yongye Huang, Jianxin Jiang, Min Wu

**Affiliations:** 1 Key Laboratory of Bioresource Research and Development of Liaoning Province, College of Life and Health Sciences, Northeastern University, Shenyang, 110169 China; 2 Wound Trauma Medical Center, State Key Laboratory of Trauma, Burns and Combined Injury, Daping Hospital, Army Medical University, Chongqing, 400042 China; 3 Wenzhou Traditional Chinese Medicine Hospital of Zhejiang Chinese Medical University, Wenzhou, Zhejiang 325000 China; 4 Wenzhou Institute, University of Chinese Academy of Sciences, Wenzhou, 325000, China; 5 The Joint Research Center, Affiliated Xiangshan Hospital of Wenzhou Medical University, Ningbo, China

**Keywords:** RNAi, Gene delivery

A recent study by Huang et al. published in *Cell* reported the application of AlphaFold2 to forecast the structures of deaminase proteins and cluster them based on structural similarity, creating a truncated Sdd that can be used as a cytosine base editor (CBE) to be integrated into a single adeno-associated virus (AAV)^1^ This ground-breaking study aided by the artificial intelligence system would largely broaden the utility of tool proteins for gene editing.

Since the one-dimensional sequence of proteins cannot fully explain their functions, the three-dimensional structure is required to provide insights into protein functions. The authors used AlphaFold2 to predict the structures of deaminase proteins. Deaminases possess critical function in host immunity against pathogens, mutation, and both DNA and RNA metabolism, and have recently been used for DNA and RNA base editors.^2^ The study discovers novel deaminases that function on single-stranded DNA (ssDNA) for CBEs, which can generate persistent C·G-T·A transversion in DNA. CBEs has primarily been developed from basic CRISPR-SpCas9 mediated gene editing to convert single targeted cytosines into thymines (C-to-T).^3^ However, only a few of ssDNA and dsDNA-targeting deaminases have been applied to construct CBEs before the present study.

The authors selected more than 200 protein sequences with deaminase domains from the InterPro database to decipher these enzymes’ structures by using AlphaFold2. They performed multiple structure alignment for all candidate proteins and generated a candidate similarity matrix based on structural similarity. They used the Unweighted Pair Group Method with Arithmetic mean (UPGMA) to organize them into a structural dendrogram, clustering the 238 proteins into 20 distinct structural branches. Each branch has different conserved protein structures. Previous studies have shown that structure-based clustering analysis is robust and effective than sequence-based clustering analysis in terms of functional similarity ranking. In summary, the artificial intelligence (AI)-assisted 3D protein structure provides reliable clustering results, which represents a convenient and effective strategy to screen deaminases.

Then, the author evaluated the editing activity of all deaminase domains by preparing each candidate domain-related sequence into CRISPR-based CBEs and co transforming them with all four fluorescent protein reporter plasmids (BFP to GFP) into rice protoplasts, and analyzed them using fluorescence microscopy. They uncovered that many deaminase branches, such as SCP1.201 (PF14428), have ssDNA cytosine deamination activity.

In previous sequence-based studies, SCP1.201 (PF14428) was named as dsDNA deaminase toxin A-like (DddA-like) deaminase in the InterPro database. The authors re-clustered it based on structure. In addition, they used a transcription activator-like effector (TALE) system to evaluate 10 proteins with similar core structures to DddA and discovered that 8 proteins my have editing activities in dsDNA base, so they named these deaminases as Ddd and classified them to a new Ddd sub-branch. To examine other SCP1.201 candidates, the researchers randomly selected 76 representative proteins from the branch and tested them in the CBE fluorescence reporter system, and 45 proteins produced fluorescence. They selected 23 protein candidates among them to evaluate the endogenous base editing efficiency via CBE mechanisms. The results of the fluorescence reporter system and high-throughput sequencing confirmed that many genes exhibit CBE activity with ssDNA, rather than dsDNA. Finally, in SCP1.201 branch, the ssDNA-targeting protein domains were named Sdds.

Most protein members in the SCP1.201 branch display structural similarity to Sdd proteins, with Sdd7 being a particularly effective ssDNA CBE. They then evaluated Sdds editing efficiency on rice protoplasts and HEK293T cells and found that Sdd7 is a powerful CBE and can be used in editing eukaryotic cells, including human and plants. Besides editing efficiency, off-targets are exceptionally important for therapeutic applications. CBEs have been found to induce substantial genome-wide off-target mutations independent of Cas9,^4,5^ therefore, the authors determined the on target/off-target ratios of 10 newly discovered Sdds. Sdd6 showed the highest on target/off-target ratios among the examined groups, indicating that some of the Sdd proteins are ideal candidates for high-fidelity base editors.

AAV delivery of CBEs holds great advantage for disease treatment, but it also has an apparent limitation: the payload space restricting CBE size. SCP1.201 deaminases have typical compactness and conservativeness, and are considered ideal proteins for single AAV CBEs. Huang et al. used AlphaFold2-assisted protein modeling to modify and shorten Sdd proteins (including Sdd7, Sdd6 and Sdd3) in size, and constructed various mini-Sdd (130~160aa). The authors applied PyMOL for multiple alignment to predict protein structure, and designed a truncated protein by removing the redundant sequence. All mini-Sdds can be used to construct single-AAV-encapsulated SaCas9-based CBEs. The higher editing efficiency and titers proved that Sdd proteins have advantages over previously tested APOBEC/AID-like deaminases (from eukaryote), demonstrating the unprecedent advantage in the AI-aided protein engineering.

The current studies have indicated that cytosine base editing at most sites in soybean crops is still challenging and inefficient. It is a situation where no base-edited soybean plant has been obtained even using the robust hA3A base editor. Excitingly, the authors obtained thirty-four gene-modified heterozygotes from 154 mini-Sdd7 gene-edited soybean seedlings. This strategy achieved high efficacy cytosine base editing to help future agricultural breeding work.

**Fig. 1:**
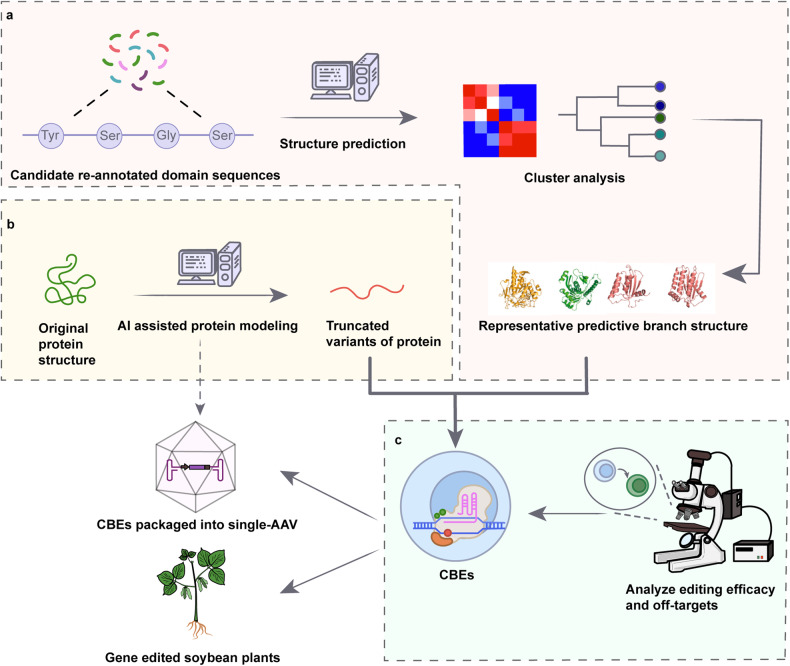
Screening deaminases with different conserved protein domains using AlphaFold2 and designing truncated deaminases using AlphaFold2 to enable efficient editing detected by the CBE fluorescence reporter system and loading into AAV for gene editing in soybean plants. **a** Protein sequences containing deaminase domains were used to predict structures using AlphaFold2 and organized into a structural tree based on structural similarity. The representative proteins from each branch node were selected for improving gene editing. **b** Reliable truncation modeling of deaminase proteins using AlphaFold2 for AAV packaging. **c** In rice and soybean, base editing with CBEs showed high on target: off-target ratios

The authors used AlphaFold2 to predict the protein structure of cytosine deaminases for structural clustering analysis, screened deaminases that can work in both plant and mammalian cells for CBEs, and used AlphaFold2-assisted design to create miniature CBEs suitable for AAV packaging. They achieved high efficacy in cytosine base engineering in soybean crops using Agrobacterium-mediated transformation for the first time, solving the problem of transgenic soybean breeding. AI prediction solves the problem that previous structural biology was hindered by the requirement of high-resolution analysis of higher structure of protein, or limited by the low precision of traditional computational-assisted folding simulations, achieving convenient and accurate prediction. In future, using AI to predict, analyze and design protein structures will help its classification research, function prediction, and directed mutation, especially for the mining and designing gene-editing tools (Fig. 1).

However, there are some limitations in this clustering approach based on AI-predicted structure alignment, which might have difficulties in classifying proteins with high sequence consistency, variable dynamic processes, and requirement for higher-precision algorithms to predict structures. In addition, the new knowledge based on AI may also raise legal and ethical issues, such as intellectual property protection, data openness and sharing, etc. In the future, AI will play an important role in disease mechanism research, genomics, epigenetics, systems biology, synthetic biology, biopharmaceuticals, and other fields.
